# Benefits of Integrating an Explicit Self-Efficacy Intervention With Calculation Strategy Training for Low-Performing Elementary Students

**DOI:** 10.3389/fpsyg.2021.714379

**Published:** 2021-08-06

**Authors:** Tuire Koponen, Tuija Aro, Pilvi Peura, Markku Leskinen, Helena Viholainen, Mikko Aro

**Affiliations:** ^1^Department of Education, University of Jyväskylä, Jyväskylä, Finland; ^2^Department of Psychology, University of Jyväskylä, Jyväskylä, Finland

**Keywords:** self-efficacy, sources of self-efficacy, math, calculation fluency, low performance, intervention

## Abstract

This study examined the malleability of math self-efficacy (SE) among children with poor calculation fluency via an intervention that targeted four sources of SE (mastery experiences, vicarious experiences, social persuasions, and emotional and physiological states). The effect of pure strategy training was contrasted with an intervention that integrated strategy training and explicit SE support. Moreover, the changes in SE source experiences and their relation with math SE, as well as the relation between math-SE profiles and calculation fluency development, were examined. In a quasi-experimental design, 60 Finnish children with calculation fluency problems in Grades 2 to 4 participated in strategy training (N = 38) or in an intervention that integrated SE support with strategy training (*N* = 32) for 12 weeks. The results showed that the explicit SE intervention integrated with strategy training enhanced math SE among children with poor calculation fluency and low SE (effect size, *r* = 0.61). Changes in mastery experiences and social persuasions were positively associated with changes in math SE among children who received the explicit SE intervention. An initially high math-SE profile and a profile indicating an increase from low to high math SE were related to growth in calculation fluency that approached the children's average age level during the interventions. In conclusion, an integrated approach that combined skill training and SE intervention was especially beneficial for children with poor calculation fluency and low math SE.

## Introduction

Self-efficacy (SE) refers to people's judgments of their capabilities to organize and execute courses of action that are required to attain designated performances (Bandura, 1986). In an academic context, SE refers to the beliefs that students hold about their capability to perform and execute a learning task under specified conditions or to perform behaviors at desired levels (Bandura, 1986). SE has been proposed to be a meaningful determinant of learning because it affects the choice of activities, effort, and persistence in learning situations (Bandura, 1986, 1997). Students who hold a low level of SE for mastering a certain task, such as in mathematics, may avoid the task or give up easily, whereas those who believe they are capable work harder and persist longer. Dysfluency in arithmetic calculation, that is, difficulty in fact retrieval, is the most typical feature of math difficulties (Geary, 1993), and it has been shown to be rather persistent (Kaufmann et al., 2011). Children with dysfluency problems often rely on slow and error-prone counting strategies despite several years of schooling (Geary, 2004); therefore, these children need to work much harder in order to complete the same number of math tasks or the same amount of homework as their typically performing peers. In order to compensate for retrieval problems, teaching efficient calculation strategies is essential. It should be noted that not only are the level of basic numerical skills or conceptual understanding in arithmetic (e.g., Canobi, 2004) related to the use of efficient counting-based calculation strategies, but self-beliefs and emotions toward math have also been shown to affect calculation efficiency (Hoffman and Spatariu, 2008). Thus, in addition to providing targeted strategy training to improve the skills, it is essential to ensure that children believe that their calculation fluency can improve with practice and that they are able to learn and use more efficient strategies. Whether children's beliefs can be strengthened by providing positive efficacy-building experiences in math still needs to be researched.

### Math Self-Efficacy and Its Relation With Math Performance

Meta-analytic studies (e.g., Richardson et al., 2012; Honicke and Broadbent, 2016) have provided empirical evidence that supports the theoretical claims of a positive correlation between academic SE and performance among middle school, high school, and college/university students. In the domain of math, several studies have shown the association between math SE and achievement among older students (e.g., Chen and Zimmerman, 2007; Ayotola and Adedeji, 2009; Kitsantas et al., 2011). There are fewer studies with younger children, but the existing literature supports the view that math SE is already related to performance in earlier stages of schooling (e.g., Pajares et al., 2007; Joët et al., 2011). In addition, most of the previous studies have focused on cross-sectional relations between math SE and math skills, and fewer studies have focused on longitudinal relations. Longitudinal studies among older students have found positive effects of math SE on later mathematics achievement (Grigg et al., 2018), an association between the growth of both constructs (Soland, 2019), and a reciprocal relation between mathematics achievement and math SE (Hannula et al., 2014; Arens et al., 2020). The few existing studies among elementary school children show more inconsistent findings. For example, Pajares and Graham (1999) found that the level of math SE predicted math performance both at the start and end of the sixth grade after taking into account motivational and emotional factors, such as self-concept and anxiety. Similarly, Galla et al. (2014) found that a higher level of academic SE was related to a faster rate of growth in math across elementary school. However, in a recent study among 4th graders (Kaskens et al., 2020) math SE did not predict the arithmetic skills in the end of 4th grade after taking into account self-concept, anxiety, and initial arithmetic skill level in the beginning of 4th grade.

Moreover, changes in academic SE (Galla et al., 2014) or in math SE (Phan, 2012b) have not been found to be related to later achievement in math, although such a relation has been found in other academic domains, such as reading (Hornstra et al., 2013; Galla et al., 2014), language (Hornstra et al., 2016), and science (Phan, 2012b). These findings from longitudinal studies raise questions regarding to what extent math SE is malleable and whether learning in math can be improved by supporting math SE in addition to skill training. To the best of our knowledge, no previous controlled intervention study has investigated the effectiveness of explicit SE intervention on math SE and math achievement.

Finally, although academic SE has been shown to be especially relevant when students encounter academic difficulties (Multon et al., 1991; Klassen, 2002), studies on math SE and math performance among elementary school children have mostly been conducted with typically achieving children, or the level of academic skills was not considered. In a meta-analysis conducted by Multon et al. (1991), the association between SE and academic performance was stronger among low-achieving students than among typically achieving students. Children with learning difficulties have also been shown to report lower general academic SE as well as lower SE in math, writing, and reading (Klassen, 2002). As low SE is assumed to decrease a student's persistence to work hard, especially when facing difficulties (Bandura, 1997), it can be especially harmful for children with learning difficulties, who need to practice harder than their typically learning peers in order to achieve required academic skills.

### Sources of Self-Efficacy

Social-cognitive theory (Bandura, 1997) hypothesizes that SE is developed and modified as children interpret information from four sources: past experiences (mastery experience), feedback and evaluative information received from others (social persuasion), models seen in reference groups (vicarious experience), and feelings and emotions experienced while engaged in or thinking about an activity or performance (physiological and affective states). However, so far there have been only a few studies that have used longitudinal or experimental designs, and thus, little understanding of the developmental dynamics between the hypothesized sources and SE.

According to Bandura (1997), the most powerful source, *mastery experiences*, stems from one's interpretations of earlier performances. For example, academic SE in a certain domain is developed and modified based on how one interprets and evaluates information about one's academic accomplishments in previous similar learning situations; experiences of success raise SE, and failures lower it. Findings from cross-sectional studies among elementary school children suggest that children rely strongly on mastery experiences when building their academic SE in different scholastic domains (see Britner and Pajares, 2006; Pajares et al., 2007; Usher and Pajares, 2008, 2009; Joët et al., 2011). However, the few existing longitudinal studies in math provide a different picture; mastery experiences have been shown to be only weakly associated with the level of math SE (Phan, 2012a,b), negatively associated (Phan, 2012a), or not at all associated (Phan, 2012b) with the growth of math SE among elementary school children when three other sources of SE were included in the same model.

The development of SE is not assumed to be dependent solely on personal interpretations of one's success in past performances but is also affected by how one experiences the feedback and support provided by others (Bandura, 1986, 1997). That is, *social persuasions* and evaluative feedback from teachers, parents, and peers influence one's SE. Younger students, in particular, have been found to depend on such feedback and have been suggested as being the most open to what others tell them, especially when learning new skills and lacking previous experience with the academic task at hand (Bandura, 1986, 1997). In particular, SE has been found to increase when students are provided with frequent and immediate feedback (Schunk, 1983). In a longitudinal study among elementary school children, social persuasions were found to be associated with the initial level of math SE but not with its growth among third and fourth graders (Phan, 2012a), whereas an opposite trend was found among fifth and sixth graders; social persuasions were associated with growth but not with the initial level of math SE. In a longitudinal study on reading SE, those students from second to fifth grade who experienced little feedback and support from teacher, parents, and peers and, more importantly, experienced loss of this social persuasion became less confident of their skills over time (Peura et al., 2021).

Moreover, learners acquire SE information through observations of models and social comparisons, that is, through v*icarious experiences*. Observing the actions of other people, such as peers and classmates, informs learners of their own capabilities (Bandura, 1997). Students who observe peers mastering a task are likely to feel more efficacious because they believe that they are also capable of accomplishing it (Schunk, 1989). Students' vicarious experiences in math can be improved by giving them the opportunity to observe their friends, who they consider to be similar to them with respect to performance level, succeed in solving problems. However, the findings from longitudinal studies are contradictory. On the one hand, a rather strong association between vicarious experiences and math SE has been found for the initial level of SE in math (Phan, 2012a), and on the other hand, no association was found with the initial level or growth of SE (Phan, 2012b). This may be partly related to the age of the children because the association was found among third and fourth graders but not among fifth and sixth graders. It has been suggested that vicarious models may play different roles as a source of math SE in different developmental stages (Ahn et al., 2017).

Students also acquire efficacy by acquiring information from their *emotional and physiological states* (e.g., anxiety, heart rate, sweating), and according to Bandura (1997), they tend to interpret physiological states as indicators of their academic competence as they evaluate their performance. A high level of negative arousal has been found to be related to lower SE in math among middle school students (Klassen, 2004) and among elementary school children (Joët et al., 2011; Phan, 2012b; Lau et al., 2018). Negative emotional states have also been shown to be negatively associated with the growth of math SE among elementary school children (Phan, 2012a,b).

A lower SE in students with learning difficulties (or learning disabilities) has been explained by them having less access to efficacy-building experiences (i.e., sources of SE) needed to develop and shape their SE (Hampton and Mason, 2003). Unfortunately, students with difficulties in learning may have fewer opportunities to experience success than their peers (Hampton and Mason, 2003; Usher and Pajares, 2006, 2008; Arslan, 2013). This would suggest that in order to boost SE among students with learning difficulties/disabilities, special attention should be placed both on the challenge level of the tasks and support in skill training as well as on feedback and activities, ensuring that they have access to all four sources of SE. Currently, there is a lack of knowledge about whether SE can be supported by providing positive source experiences, and especially so among children with learning difficulties/disabilities.

### From Theory and Empirical Evidence to Intervention

Despite the strong theoretical framework of socio-cognitive learning and some empirical evidence that supports the association between SE and its four sources, few intervention studies have aimed to enhance SE by enabling positive source experiences and examining the influence of SE level and its changes on the development of academic skills. Interventions among children with learning difficulties have mainly focused on providing training for the compromised skill itself or the cognitive skills assumed to underlie the academic difficulties (Kearns and Fuchs, 2013). The intervention studies that have aimed to enhance participants' SE in math have focused on strategies and goal-setting instructions (Schunk, 1985) together with social comparative information (e.g., Schunk, 1983) and peer models (Schunk and Hanson, 1985). To the best of our knowledge, there are no previous SE intervention studies among poor-performing children in the context of math that explicitly target all four sources of SE, even though the social-cognitive theory hypothesizes that SE beliefs are developed and modified as children interpret information from the four sources. In addition, it has been shown that intervention effects on SE become larger as the number of sources included in the intervention increase (Unrau et al., 2017). Covering all four sources in the interventions is further supported by findings that indicate that at the individual level, students may rely on different sources of information in varying combinations (Chen and Usher, 2013). Furthermore, students with learning difficulties are assumed to have less access to sources of efficacy information (Hampton and Mason, 2003), and low-performing students have been shown to lose source experiences over time, and this has been found to relate to their decreasing self-efficacy (Peura et al., 2021). Thus, if we assume that exposure to sources of SE enhances SE and thereby positively influences effort and persistence in learning situations and consequently learning, it would be of utmost importance to provide positive source experiences, especially for students with learning difficulties or low performance.

To our knowledge, only two intervention studies have targeted all four sources of SE (mastery experiences, social persuasions, vicarious experiences, and the psychological and affective state) among elementary school children with learning difficulties or low achievement. One focused on writing skills (García and de Caso, 2006), and the other focused on reading fluency (Aro et al., 2018). The findings of these two studies have been encouraging. García and de Caso (2006) found that writing skills can be improved by enhancing children's writing SE with the establishment of a positive psychological and affective climate, created by providing social persuasions, explicating mastery, and providing vicarious experiences. Aro et al. (2018), in turn, found that intervention resulted in a greater positive change in reading SE in the group that was provided explicit SE support in addition to reading fluency training than in the group that was only provided reading fluency training. Moreover, a change in reading SE was positively associated with a change in reading fluency only within the group that received explicit SE support. In the target skill itself, reading fluency, the two intervention groups showed equal improvement.

### Present Study

The purpose of the present study was to fill existing gaps in the field of math-SE research and examine to what extent explicit SE support that incorporated targeted skill training supports SE, as compared to targeted skill training only.

The present study had three aims. The first aim was to examine the changes in math SE among two calculation strategy training groups, one with (*the SE group*) and another without (*the skill group*) explicit SE intervention, and their controls. The second aim was to examine the changes in the source experiences of SE during the intervention among children participating in two interventions (the SE and skill groups) and the relation between the changes in the source experiences of SE and math SE. The third aim was to examine the association between different SE profiles formed on the basis of the level and change in math SE during the intervention (i.e., high SE, low-to-high SE, low SE, and low-increasing SE) and improvement in calculation fluency during the intervention.

In the present study, we extended a recent study with the same participants, which focused on reporting the effectiveness of calculation strategy training on calculation fluency among second to fifth graders who used immature counting-based strategies in basic addition despite formal schooling for several years (Koponen et al., 2018). In that study, strategy training was shown to be effective in supporting calculation fluency; both intervention groups receiving the identical strategy training (with and without SE intervention, SE and skill groups) showed improvements in calculation fluency during the intervention, outperforming the control groups that received either the corresponding intervention in reading (children with low reading fluency) or business-as-usual support for math at schools. However, in that study, the changes in math SE or source experiences were not examined. In the present study, the following specific research questions were addressed:

#### Math Self-Efficacy

- *To what extent does explicit calculation strategy training with or without additional explicit math-SE intervention enhance math SE?* Changes in math-SE were compared between the two intervention groups (SE and skill) and with the business-as-usual controls.
- *To what extent does explicit calculation strategy training with and without explicit math-SE intervention enhance math SE among children with an initially low math SE?* Children reporting a low pre-intervention math SE were included in these analyses in order to study the influence of the intervention conditions among children in most need of support for math SE.

#### Source Experiences and SE in Math

- *To what extent does explicit calculation strategy training with and without explicit math SE enhance source experiences?*
- *Are the changes in sources related to changes in math SE during the interventions?*

#### Self-Efficacy and Skill Development

- *Are the level and changes in math SE related to improvement in calculation fluency, that is, are there differences between children with different SE profiles (i.e., high SE, low-to-high SE, low-increasing SE, and low SE) in calculation fluency change?*

## Method

### Participants

This study was part of a longitudinal research project (Self-efficacy and Learning Disability Intervention (SELDI; 2013-2015)) that focuses on elementary school children's self-beliefs, motivation, and reading and math fluency skills. The data for the present study were collected over two consecutive autumn terms, with the first measurement point in November and the last one in October of the next school year.

A total of 20 schools in urban and semi-urban areas in Central and Eastern Finland volunteered to participate, from which the classes and children were recruited for this study to implement calculation or reading fluency interventions. Ten of the schools provided calculation fluency interventions. Written consent was obtained from the guardians of the participants. The research procedure was evaluated by the University of (Jyväskylä) Ethical Committee.

The original sample consisted of 1,327 children (638 girls, 689 boys) from Grades 2 to 5. Of this sample, 178 (13.41% of the original sample) were second graders (*M*_*age*_ = 8.35 years, *SD* = 0.32 years), 471 (35.49%) were third graders (*M*_*age*_ = 9.34 years; *SD* = 0.31 years), 383 (28.86%) were fourth graders (*M*_*age*_ = 10.40 years; *SD* = 0.35 years), and 295 (22.23%) were fifth graders (*M*_*age*_ = 11.39 years; *SD* = 0.36 years). After screening this larger sample using at or below the 20th percentile as a criterion for poor performance, 240 children were screened for individual assessment in calculation fluency; after which, 69 children were selected to participate in calculation strategy training (see the description of the screening process below) with (SE group) or without (skill group) an explicit self-efficacy intervention. In addition to the confirmed weakness in calculation fluency (use of counting-based strategies), the project's parallel reading interventions and available resources for special education defined the number of final intervention groups, and thus, the number of children participating in the calculation fluency intervention. Intervention was provided mainly for children from second to fourth grades, but some fifth graders were included as well. To form a control group (*N* = 69), one child from the class of each participant in the math intervention was selected based on having the next-lowest calculation fluency score. Classmate controls were matched for gender (when possible), and they received business-as-usual support, including any special education usually provided in the school. Controls who came from the same classes as the children in the SE group did not differ from those controls that came from the same classes as children in the skill group in the improvement of self-efficacy or calculation fluency during the intervention period (*p* > 0.05), and thus, they were combined to form one control group. The two intervention groups and the control group did not differ in age or non-verbal reasoning (Raven's Matrices test, *p* > 0.05). The two intervention groups were matched with the initial level of calculation fluency, and they did not differ in the initial level of math SE (*p* > 0.05).

A quasi-experimental design was applied. The schools, classes, and teachers volunteered to participate, and the caregivers gave written consent for participation. The study was carried out at the participating schools and during regular school hours. Screening was conducted with regard to both reading and calculation fluency, and the volunteering schools selected for calculation interventions were randomized to have the calculation strategy training either with or without specific SE intervention. This was done in order to avoid treatment contamination, which could happen if the programs were provided in the same school. Approximately half of the children participating in the calculation intervention received SE intervention following a manual-based intervention program, and the other half participated in groups in which the teachers were not explicitly instructed with regard to SE but were provided a manual-based strategy training program. This design insured that the groups had identical strategy training. There were no differences between the two intervention groups in terms of calculation fluency in the pre-intervention assessments.

#### Screening Procedure for Intervention

Screening for inclusion in the calculation strategy intervention was carried out in two steps. First, all participants from the original sample were assessed in terms of their calculation fluency using group-administered timed calculation tasks. Children from Grades 2 to 4 whose performance was at or below the 20th percentile in the calculation fluency task (compared to their grade level) were selected for individual assessment, which included 20 single-digit addition items (e.g., 2+8, 5+4, 9+6, 7+3) presented one by one in a game-like context. The children were asked to respond as quickly as possible to each item. A point was given for correct responses within 3 s. Inclusion criteria for the intervention were that the children showed dysfluency, both in the group-administered calculation fluency task (i.e., performance at or below the 20th percentile) and in the individual assessment situation that required fast fact retrieval or the efficient use of back-up strategies (slow or incorrect responses on at least 30% of the simple addition items). Out of the 240 children who in the group administered calculation fluency task showed calculation fluency below the 20th percentile, two children had missing data, and in the individual assessment situations, 154 children also showed use of immature calculation strategies. Eight of these children with dysfluency in reading also participated in the reading intervention. Altogether, 69 of the children who met the criteria and were from those schools implementing calculation intervention were included in the present study (77 children were from schools and classes where math intervention was not implemented). Additionally, six children with low calculation fluency but who did not meet the selection criteria participated in the calculation intervention for practical reasons (i.e., to be able to form a group at the school) and were not included in the analyses. The number of children receiving SE intervention embedded in strategy training was 31 (SE group), and 38 children received just strategy training (skill group). The final groups for this study were composed of the children for whom there were complete SE data from all four assessment points: 28 children in the SE group and 32 in the skill group. Children who had missing data did not differ from those children who had full data in the initial level of calculation fluency. The main reasons for missing data were absences from school on assessment days or moving to another school.

#### Intervention Design and Procedure

We applied an intervention design with two pre-assessments, one post-assessment, and one follow-up assessment as a part of a larger longitudinal follow-up study. Pre-intervention assessments were conducted in November and January. The 12-week interventions started at the end of January. A post-intervention assessment was conducted after the intervention ended in April, and a follow-up assessment was performed 5 months after the intervention, at the end of September or the beginning of October. At the second pre-intervention assessment, a shortened assessment battery, including addition and subtraction fluency tasks, was administered during one group assessment session. The group assessment was administered before the individual assessment at each time point.

### Measures

#### Calculation Fluency Measure

Basic addition fluency was assessed using a group-administered paper-and-pencil test with 120 items and a 2-min time limit (Koponen and Mononen, 2010). The addends had values of 10 or smaller. One point was given for all items answered correctly within the time limit, and the total score was calculated. Correlations with other calculation fluency tasks (subtraction and arithmetic tasks with multiple operations) varied from 0.74 to 0.85.

An individually administered addition fluency task was used for screening children with low calculation fluency (at or below the 20th percentile) to confirm that the dysfluency in calculation was real and not due to other factors that were not possible to detect in the group assessment (for details, see Koponen et al., 2018). The individual game-like assessment used a *no-choice technique* to assess addition fluency. The children were shown a card with an addition problem on it and were required to answer correctly within 3 s to win the card. One point was given for all items answered correctly within the time limit, and the total score was calculated.

#### Math SE

The group-administered questionnaire specifically targeting math-SE was developed based on the guidelines outlined by Bandura (2006). Researchers with expertise in self-efficacy, math development and learning difficulties were consulted in item formulation. Two different specificity levels of self-efficacy for arithmetic were assessed: intermediate, and general level. Items targeted arithmetic skills, learning and applying the skills in daily settings, and thus were appropriate and concrete for primary school children (see Appendix A). The children completed the questionnaire before the calculation fluency assessment. Trained research assistants gave pre-written instructions and read aloud all the questionnaire items one by one to ensure that everyone could answer them regardless of their reading skill. The items began with the question “*How certain are you that you can.,”* and the children rated the strength of their confidence using a seven-point scale ranging from “*I'm totally certain I can't.”* (1) to “*I'm totally certain I can.”* (7). The questionnaire covered seven self-efficacy items that were related to calculation skill: beliefs on one's current ability in calculation (two items), one's ability to learn to be more fluent/accurate (two items), and one's ability to apply calculation skills in daily life (three items). The items are presented in Appendix A. Cronbach's alpha for the self-efficacy scale was 0.71.

To examine the association between SE and fluency improvement during the intervention (RQ3), all the intervention children were classified into four groups according to their ratings on the SE questionnaire before and after the intervention. The cut-off score for the low SE group (at or below 42 points) was based on the whole sample using the median score for math SE for the low-performing children (at or below the 20th percentile in addition fluency; *N* = 263). The high SE group (*N* = 15) included children whose total SE score was above 42 before and after the intervention. The low-to-high SE group (*N* = 16) included children whose SE score was at or below 42 but was above 42 after the intervention. The low-increasing SE group (*N* = 9) scored at or below 42 both before and after the intervention but showed SE enhancement during the intervention. The low SE group (*N* = 18) included the rest of the children with an SE score at or below 42 both before and after the intervention and without enhancement in SE.

#### SE Source

Sources of math SE were assessed using 12 items, adapted from a questionnaire previously validated by Usher and Pajares (2009). Children rated their mastery experience (three items, e.g., “I do well in math”), social persuasions (three items, e.g., “My teacher has often told me that I am getting better in math”), vicarious experience (three items, e.g., “I admire adults who are good in math”), and physiological and emotional state (three items, e.g., “I feel tension when I have to do math”*)* using a 7-point Likert scale (1, *not true*, to 7, *true*). The items are presented in Appendix B. Higher scores for mastery experience, social persuasions, and vicarious experience referred to positive experiences, whereas higher scores on the physiological and emotional state subscale represented experiencing more adverse physiological arousal and emotional states (reverse scoring was used in the total score). Cronbach's alphas for the source experience scales were 0.86 at pre-assessment 2 and 0.77 at post-assessment.

### Intervention Programs

#### Calculation Strategy Training

In the present intervention study, both intervention groups received a similar type of calculation strategy training implemented based on a shortened version of the SELKIS intervention program (Koponen et al., 2011). This program focuses on derived fact strategy training and aims at helping children to discover more efficient calculation strategies using their existing knowledge of number sequences, number concepts, and arithmetical facts (conceptual knowledge). Addition fluency was selected for the training context in math because it forms a ground for other arithmetic operations, such as subtraction and multiplication, which are even more difficult and laborious to solve using only counting-based strategies. Children participated in the strategy training group sessions twice a week for 45 min at a time. The number of participants in the groups varied between four and six. In addition, they had two short weekly gaming sessions for practicing basic addition skills by playing math games and received a worksheet for homework that included similar types of addition problems practiced during strategy sessions (for details, see *Authors*).

#### SE Intervention

The intervention elements (see Table 1) aimed at enhancing math SE explicitly targeted the four sources of SE (Bandura, 1997). *Mastery experiences* were provided by using individually challenging but accessible tasks. This element was also present in the skill program, but in the SE program only, several forms of feedback and practice were provided to insure that each individual's progress became visible, thus assuring mastery experiences. First, positive, explicit, and concrete feedback was provided on improvements in calculation fluency and on shifts toward using more efficient calculation strategies. During the 12-week intervention, children practiced four sets of addition problems, and before and after training on each set of problems, they carried out a 1-min calculation fluency task. Based on the results (the sum of correctly solved problems), they were allowed to color the corresponding number of floors on a tower. Attention was paid to each individual's improvement. Second, twice a week, the children participated in short game sessions in which they practiced calculation strategies by playing math games. The sessions were guided by a school assistant (or class teacher). In the SE program, school assistants were trained to give feedback related to a child's improvement compared to his/her previous performance or the effort she/he showed during the game session. In both intervention programs, children received a sticker or stamp after each game session indicating attendance. To provide children with *social persuasions*, the teachers in the SE group explained and verbally praised the children's efforts in practicing and improvement. Particular attention was paid to the children's development and effort, but the reasons for temporary setbacks were also discussed. In the SE group, the teacher started each intervention session by providing verbal feedback related to the homework tasks (reminding them of the importance of training and effort, etc.), and each child shared with the teacher the feedback he/she received from the school assistant in the game session, which was written on a game pass. In the skill group, the teacher was instructed to check homework regularly. Moreover, during the SE intervention, teachers had private discussions with each child that focused on what types of strategies she/he used before the training and how the distribution and frequency of the strategies changed during the training. The teacher demonstrated to each child his/her progress in applying more efficient calculation strategies using a picture of stairs to visualize the strategy development. In addition to the social persuasions from the teachers and school assistants, the children were also encouraged to provide positive feedback for each other related to the use of fluent calculation strategies or signs of improvement.

**Table 1:** Intervention structure: Weekly SELKIS-intervention sessions and game sessions and elements of the SE-program to foster self-efficacy.

	**SKILL-program**	**SE-program**
**Time used**	**Game sessions**	
15 min two times per week	Math games guided and attendance marked by school assistant or regular class teacher	Math games guided and SE feedback given by school assistant or regular class teacher
**Time used**	**Weekly SELKIS-intervention sessions**	
5 min	Welcome and orienting	Welcome, orienting, and emotion checklist
5 min	Checking homework	Sharing feedback from the last game session, checking homework, and giving SE feedback
25–30 min two times per week	SELKIS-strategy training	SELKIS-strategy training integrated with SE intervention (feedback on effort, personal progress, and use of fluent strategies, encouraging peers, stories and discussion related to learning, emotion, and SE)
5 min	Cleaning up, homework	Cleaning up, homework, emotion checklist
	**Sources of self-efficacy provided during the weekly group sessions**	
Mastery experience	•Reachable challenges with exercises adapted to each child's skills	•Reachable challenges with exercises adapted to each child's skills •Individual concrete visual feedback on progress in calculation fluency (e.g., calculation fluency towers, •Individual concrete feedback on improvement in the use of efficient calculation strategies (e.g., stairs describing the development of calculation strategies) •Individual concrete feedback on working habits and effort during and after each group session, game session, and homework (e.g., discussions)
Vicarious experience	•Exercises in a peer group with similar skill levels	•Exercises in a peer group with similar skill levels •Mastery models observing peers and focusing on good performance and improvement of the peers
Verbal persuasion		•Systematic feedback on development and effort verbalized by teacher •Encouraging feedback from peers •Directing child's attention to his/her own improvement and recognizing it
Affective reactions		•Naming of affective state, discussions on emotions concerning learning and self-ratings of willingness to practice •Stories and discussion about the relation between emotion, thoughts, behavior, and learning •Mistakes and setbacks accepted and allowed in a positive atmosphere •Filling in the emotional checklist at the beginning and at the end of the session

To assure *vicarious experiences*, the children worked in groups with similar levels of calculation fluency. The participants in the SE program were also encouraged by the teacher to observe the improvements of their peers and share these with the group to provide vicarious experiences. For example, participants were encouraged to point out efficient strategies used within a group at any time during the sessions. After identifying an efficient strategy, they colored one circle of a strategy chain that was visible in the classroom during the intervention sessions to demonstrate concretely that they were making progress as a group. Moreover, they played a card game in which each participant had a pile of cards with addition problems; they had to solve as many problems as possible within 1 min and mark down their score. Other participants provided encouragement (*social persuasions*). After the first round, all the scores were totaled for the team score. The aim for the second round was to beat one's own first score and together with the other participants to help obtain a better team score, reflecting progress and success as a group.

To think about and discuss the *emotions related to learning and practicing*, the participants filled in an emotional checklist indicating how eager they were to practice. These self-ratings were completed at the beginning and at the end of each session to enable discussions about learning-related emotions and to provide an opportunity to express feelings about the strategy training. The SE program included stories related to emotions, beliefs, choice of actions, and consequences in learning that were read by the teacher and discussed together with the children. Using the self-ratings and discussions, the aim of the SE program was to enhance awareness as well as self-knowledge of how emotions and beliefs influence one's behavior in learning situations and learning outcomes.

### Teacher Training and Fidelity

Before the intervention, the researchers instructed all the participating teachers on how to implement the program for the calculation strategy training. Moreover, the teachers who conducted the strategy training with self-efficacy feedback were shown how to provide feedback and implement group activities aimed at supporting self-efficacy in math. All the teachers in both groups received group-specific, detailed session-by-session manuals. Two 3-h training sessions were organized, which included the theory of calculation fluency development and how to implement intervention in practice using the program manual.

A number of methods were used to ensure the fidelity of the interventions. First, the teachers were trained in small groups so the instructions for the interventions could be delivered separately for each intervention program. Second, the teachers were provided with session-by-session manuals and materials. Third, meetings and telephone conversations were arranged to monitor adherence to the intervention protocols; after the third intervention session, researchers called each teacher to ensure that the manuals were followed and that the main principals of the programs were understood. Moreover, two meetings were arranged during the interventions to share experiences and ensure that all teachers understood the key elements of the intervention. Fourth, teachers were given a checklist of the feedback to provide for each child on improvement, the amount of work done, effort, and persistence during the practice. The teachers also completed a checklist diary, marking the completed intervention sessions and noting any exceptions in intervention activities or the attendance of participants. Finally, at the end of the intervention, a questionnaire was completed by the participating children in order to check that their experiences with the practices within the interventions corresponded to the intended content. The questionnaire consisted of 28 items with a four-point scale ranging from “*Always…”* (1) to “*Never”* (4). The questions asked about the feedback and evaluations the child felt she/he had received from the teacher on his/her improvement compared to his/her performance at the beginning (making the progress visible to children; mastery experience), social persuasions and feedback given by teacher on training and trying hard (social persuasions), social persuasions and feedback given by other group members (social persuasions), observing the improvement of others in the group (vicarious experience), discussions on emotions and thoughts regarding learning (emotions/thoughts), and questions about more general issues concerning the intervention atmosphere and content (general). Total scores were calculated for each scale. The skill and SE groups differed significantly on all the scales concerning SE-specific content (the Mann-Whitney U test showed significant *p*-values that varied from 0.029 to 0.001), as the SE children reported more SE-related source experiences. In contrast, no difference was detected in the general scale (*p* > 0.05). These differences imply that the interventions were perceived differently by the children in all aspects relevant to explicit SE support in math.

There were 128 activities within 24 intervention sessions (introduction of strategies, games/exercises, starting and closing activities), and the average proportion of activities completed by teachers without exceptions (e.g., did not have time enough) was 97%. The attendance percentage of individual children typically varied between 92 and 100% in a group, meaning that in most of the groups, a child was absent for no more than two of the 24 intervention sessions. However, there were four children who missed four out of 24 intervention sessions, one missed five sessions, and one missed seven sessions. All of these children were included in the analyses.

### Data Analyses

Due to the relatively small sample sizes and non-normally distributed SE variables (Kolmogorov-Smirnov, *p* < 0.05), non-parametric analyses were used for the first research question. To analyze the intervention effects on SE, the within-group changes in SE over the three time periods (baseline, intervention, and follow-up) were analyzed separately for the two intervention groups and the control group by using a non-parametric Friedman test, and for the *post-hoc* analysis, the Wilcoxon signed-rank test with Bonferroni correction was used. In addition, comparisons of the SE gain scores during the intervention were conducted using the Kruskal-Wallis test and pairwise comparisons using the Bonferroni approach. Moreover, the same analyses were rerun with only the children who had low SE before the intervention (cut-off score for low SE at or below 42 points).

Second, analyses related to changes in source experiences during the intervention and association with SE were conducted for the two intervention groups. The source variables were mainly normally distributed (Kolmogorov-Smirnov, *p* > 0.05), and parametric analyses were used. However, for two source variables (vicarious experiences and emotional and psychological states) with non-normal distributions, additional analyses were conducted using non-parametric methods. The development during intervention was analyzed by using repeated measures ANOVA and the Friedman test. The comparison of the gain scores during the intervention was tested by using an independent sample *t*-test and Mann-Whitney U test. The association between changes in source scores and SE were analyzed by using Spearman's rank-order correlation coefficient.

Third, in order to analyze the influence of the level and changes in SE on skill development, all children from the two intervention groups were classified into four SE profiles based on their SE ratings before and after the intervention (high SE, low-to-high SE, low SE, and low-increasing SE). The change in calculation fluency was analyzed by using the Friedman test, and for the *post-hoc* analysis, the Wilcoxon signed-rank test with Bonferroni correction was used. In addition, a comparison of calculation fluency gain scores between the profile groups was conducted using the Kruskal-Wallis test.

Changes in the target variables (SE or calculation fluency) during all possible time periods between the four assessment points were analyzed in RQ1 and RQ3, and the Bonferroni correction took these multiple comparisons into account. However, here we report only the results from the periods that were relevant for the intervention design: between pre-assessments 1 and 2 (baseline), between pre-assessment 2 and the post-assessment (intervention), and between the post-assessment and follow-up assessment (follow-up).

Effect sizes, *r* = Z/√(N), were computed from standardized test parameters (Field, 2013), and the partial eta squared was reported for the ANOVA models. The following intervals for *r* were used according to Cohen (1988): no effect, < 0.1; small effect, 0.1–0.3; intermediate effect, 0.3–0.5; and large effect, >0.5. Corresponding intervals for the partial eta squared were as follows: no effect, < 0.01; small effect, 0.01–0.09; intermediate effect, 0.09–0.25; large effect, >0.25. Effect sizes were used as a parallel source when considering the strength of the evidence, and unlike the *p*-value, it is independent of sample sizes.

## Results

### RQ1: Effects of Calculation Strategy Training and Explicit SE Intervention on Math SE

First, we analyzed the within-group changes in math SE over the four assessment points (Table 2). Math SE was found to change in all three groups (SE, skill, and control). *Post-hoc* analysis revealed a significant increase in math SE among both intervention groups but not among the control group during the intervention. The effect size was intermediate in the SE group and small in the skill group (Table 3). After taking into account the Bonferroni correction, a significant adjusted *p*-value was found only for the SE group. None of the groups had significant changes in math SE during the baseline or follow-up period. A closer examination of the changes for those children who had low math SE before the intervention revealed that only in the SE group did the children with initially low math SE show improvements in their math SE during the intervention period. The effect size was large (Table 3).

**Table 2:** Changes in self-efficacy among two intervention groups and controls (RQ1).

**Group**	**Scores at each assessment points**					**Friedman test**	**Paired comparison**		
		**Pre1**	**Pre2**	**Post**	**Follow-up**		**Pre1 vs. Pre2**	**Pre2 vs. Post**	**Post vs. Follow-up**
SE group	*Md*	42	41.5	44.5	44	16.95^**^ (3, 28)	0.05	−2.85^**^	0.16
	*min/max*	*25/46*	*16/49*	*32/49*	*30/49*			(adj. *p* = 0.027)	
Skill group	*Md*	38.5	39.5	41.5	42	9.68^*^ (3, 32)	−0.1	−2.23^*^	0.29
	*min/max*	*15/49*	*21/48*	*20/49*	*18/49*			(adj. *p* = 0.121)	
Controls	*Md*	44	45	46	46	9.11^*^ (3, 51)	−1.38	−0.27	−1.15
	*min/max*	28/49	28/49	27/49	31/49				
SE group_low_	Md	36	36	42	43	23.35^***^ (3,13)	−0.15	−3.11^**^	−0.3
	*min/max*	*30/42*	*16/42*	*32/49*	*36/49*			(adj. *P* = 0.011)	
Skill group_low_	Md	31	37	37	40	8.09^*^ (3,17)	0.18	−1.2	0.27
	*min/max*	*15/42*	*21/42*	*20/47*	*18/49*				
Controls_low_	Md	37	33.5	37.5	36	4.55 (3, 12)	NA	NA	NA
	*min/max*	*29/42*	*28/40*	*27/48*	*31/49*				

* *p < 0.05,*

** *p < 0.01,*

*** *p < 0.001.*

*Low refers sub group of children having self-efficacy level at or below 42 points at pre assessments. Md, median score; min, minimum score; max, maximum score*.

**Table 3:** Effect sizes for changes in self-efficacy (RQ1).

**Group**	***N***	**Effect sizes (** ***r*** **)**		
		**Pre1 vs. Pre2**	**Pre2 vs. Post**	**Post vs. Follow-up**
**SE group**	28	0.01	0.38	0.02
**Skill group**	32	0.01	0.28	0.04
**Controls**	51	0.14	0.03	0.11
**SE group**_**low**_	13	0.03	**0.61**	0.06
**Skill group**_**low**_	17	0.03	0.21	0.05
**Controls**_**low**_	12	NA	NA	NA

*Large effect sizes are written in bold. Following intervals for r were used according to Cohen (1988): no effect < 0.1; small effect 0.1–0.3; intermediate effect 0.3–0.5; >0.5 large effect. Low refers sub group of children having self-efficacy level at or below 42 points at pre assessments*.

Second, changes in math SE during the intervention (SE-gain score) were compared among all three groups. The Kruskal-Wallis test revealed significant differences among the groups (Table 4). Pairwise comparisons showed significant differences between the SE group and the controls as well as between the skill group and controls; the intervention groups had a larger increase in math SE during the intervention compared to the controls. After taking into account the Bonferroni correction for multiple tests, the adjusted *p*-value indicated significant differences between the SE group and the controls (*p* = 0.02) but not between the skill group and the controls (*p* = 0.08). A closer examination of the SE-gain scores of those children who had low math SE before the intervention revealed a value close to the alpha level of 0.05 (*p* = 0.067) but that was deemed not significant by that standard. A pairwise comparison using the Mann-Whitney U test between the children with low math-SE in the two groups revealed a higher increase (intermediate effect size) in math SE among the SE group than in the skill-group participants (*U* = 162.00, *Z* = 2.16, *p* = 0.031, *r* = 0.39). The difference in the increase was close to the pre-set alpha level (but not achieving it) when the SE group was compared with the control group (*U* = 45.50, *Z* = −1.83, *p* = 0.068, *r* = 0.36); however, the effect size was intermediate. The skill and control groups did not differ from each other, and effect sizes indicated no effect (*U* = 104.50, *Z* = 0.11, *p* = 0.913, *r* = 0.02).

**Table 4:** Comparison of gain scores in self-efficacy during intervention (RQ1).

	**Groups**									**Test**
	**SE**			**Skill**			**Control**			**Kruskal Wallis Test**
	**M (sd)**	**Md**	**min/max**	**M (sd)**	**Md**	**min/max**	**M (sd)**	**Md**	**min/max**	
Self-efficacy gain score (Post-Pre2)–all	4.86 (8.98)	2.50	−15/33	2.60 (4.56)	2.00	−7/15	0.53 (5.13)	0	−10/21	9.16^**^ (2, 111); SE = Skill > controls
Self-efficacy gain score (Post-Pre2)–low	9.23 (9.82)	7.00	−3/33	2.58 (4.75)	1.00	−4/15	2.50 (6.02)	4.00	−8/15	5.39 (2, 42)

** *p < 0.01. Low refers sub group of children having self-efficacy level at or below 42 points at pre assessments*.

### RQ2: Effects of Interventions on Source Experiences and the Relation Between Changes in Source Experiences and in Math SE

The changes in the sources of math SE (total score, mastery experiences, social persuasions, vicarious experiences, and emotional and physiological states) were analyzed by using repeated-measures ANOVA with time (pre-test1 vs. pre-test2 vs. post-test vs. follow-up) as a within-subject factor and *intervention* group as a between-subjects factor. A significant main effect of time was found, indicating that children's source experiences increased during the intervention when analyzing an overall total score [*F*_(1, 58)_ = 5.44, *p* = 0.023, $\eta_{p}^{2}$ = 0.09] and in specific types of sources of mastery experiences [*F*_(1, 58)_ = 4.56, *p* = 0.037, $\eta_{p}^{2}$ = 0.07] and emotional and psychological states [*F*_(1, 58)_ = 4.59, *p* = 0.036, $\eta_{p}^{2}$ = 0.09] across the sample. Moreover, there was a significant interaction between time and group in social persuasions [*F*_(1, 58)_ = 5.43, *p* = 0.023, $\eta_{p}^{2}$ = 0.09], indicating that social persuasion experiences strengthened in the SE group and decreased in the skill group. Due to non-normal distribution findings related to sources of vicarious experiences and emotional and psychological states, the results were confirmed by using a non-parametric Friedman test. The findings of non-significant changes in vicarious experiences during the intervention were fully supported by the non-parametric analyses (χ^2^ = 0.78, df = 1, *p* = 0.736), and the results related to emotional and psychological states were in line with the parametric analyses, although they did not reach a significance level of 0.05 (χ^2^ = 2.81, df = 1, *p* = 0.093). The comparisons in source gain scores during the intervention were conducted by using an independent sample *t*-test and confirmed by the Mann-Whitney U test for sources of emotional and psychological states and vicarious experiences. The only difference was found in the gain scores of social persuasions favoring the SE group [t(58) = −2.53, *p* = 0.014].

The association between the gain scores in the source and math SE (during the intervention) were analyzed by using Spearman's rank-order correlation coefficient (Table 5). Changes in math SE among the SE-group participants were correlated with changes in mastery experiences (*r*_S_ = 0.48, *p* = 0.010) and social persuasions (*r*_S_ = 0.43, *p* = 0.024). The correlation between the other two sources and math SE varied from small (vicarious experiences) to intermediate (emotional and psychological states); the correlation did not reach the pre-set level of significance with the emotional and psychological states (*p* = 0.077). Changes in the source experiences and math SE were not related among the skill-group participants, as the correlations were generally very low, varying mainly from 0.09 to 0.18. The only exception was the association between changes in mastery experiences and math-SE, which was at the intermediate level and close to the pre-set level of significance (*r*_S_ = 0.32, *p* = 0.085).

**Table 5:** Spearman's rank-order correlation coefficient between gain scores in source experiences and in self-efficacy among SKILL-group and SE-group (RQ2).

	**1**.	**2**.	**3**.	**4**.	**5**.	**6**.
Self-efficacy (gain score)	-	0.48^*^	0.43^*^	0.24	0.34	0.29
Mastery experiences (gain score)	0.32	-	0.31	0.35	0.11	0.62^***^
Social persuasions (gain score)	0.09	0.22	-	0.20	0.23	0.43^*^
Vicarious experiences (gain score)	0.18	0.32	0.32	-	0.00	0.66^***^
Emotional and physiological states (gain score)	0.10	0.06	−0.08	0.19	-	−0.37
Source experiences (gain of sum score)	0.18	0.60^***^	0.49^**^	0.56^**^	−0.31	-

* *p < 0.05,*

** *p < 0.01,*

*** *p < 0.001. Correlations for SKILL-group are below the diagonal; correlations for SE-group are above the diagonal*.

### RQ3: Differences in Math SE and Changes in Calculation Fluency During the Intervention

**Figure 1:**
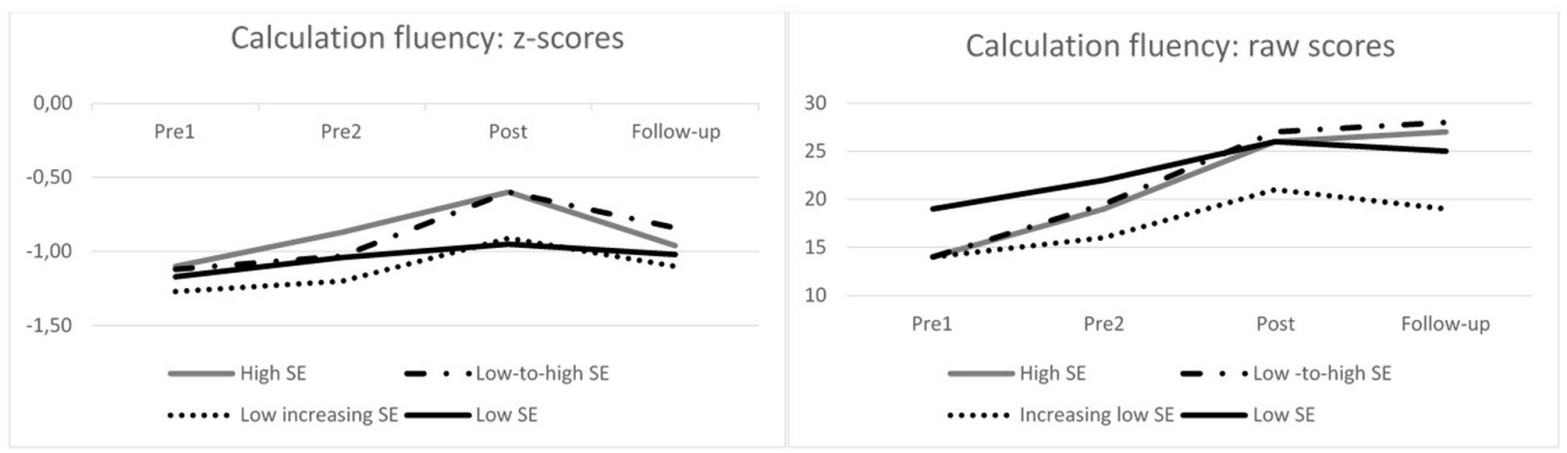
Development of calculation fluency among children with different SE-profile.

In order to analyze the influence of the level and changes in math SE on skill development, first the within-group changes in math SE among children with different SE profiles (high SE, low-to-high SE, low-increasing SE, and low SE) were analyzed over the four assessment points (Figure 1). A non-parametric Friedman test of differences among the repeated measures was conducted separately for each group, both with raw scores (absolute change) and standardized scores (adjusted with the average grade level and variation) (Table 6). Changes in the calculation fluency during the assessed time period were found for all groups when analyzing raw scores, and for the high SE and low-to-high SE profiles when analyzing z-score changes. Moreover, a *post-hoc* analysis with the Wilcoxon signed-rank test showed that when using raw scores, there was a significant change in calculation fluency during the intervention in the high SE, low-to-high SE, and low-SE profile groups and a close but not significant change (*p* = 0.068) in the low-increasing SE group. The effect sizes (*r*) for changes during the intervention were large for the high SE group and intermediate for the other three SE groups (Table 7). None of the groups showed significant development during the baseline or follow-up (*p* < 0.05). When analyzing the z-scores, the high SE-group and low-to-high SE-group approached their average grade level in calculation fluency during the intervention but not during the baseline or follow-up. The size of the improvement was large for the high SE group and intermediate for the low-to-high SE group.

**Table 6:** Changes in calculation fluency among children with different self-efficacy profile (RQ3).

**Group**	***N***	**Score**	**Calculation fluency**					**Friedman test**	**Paired comparison**		
				**Pre1**	**Pre2**	**Post**	**Follow-up**		**Pre1 vs. Pre2**	**Pre2 vs. Post**	**Post vs. Follow-up**
High SE	15	Raw	*Md*	14	19	26	27	33.53^***^ (3, 15)	−1.49	−3.47^**^ (adj. *P* = 0.003)	0.64
		Z-score	*Md*	−1.1	−0.87	−0.6	−0.96	18.04^**^ (3, 15)	−0.85	−3.11^**^ (adj. *P* = 0.011)	1.70
Low-to-high SE	16	Raw	*Md*	14	19.5	27	28	30.75^***^ (3, 16)	−1.85	−2.47^*^ (adj. *P* = 0.082)	−0.48
		Z-score	*Md*	−1.12	−1.03	−0.6	−0.84	16.64^**^ (3, 16)	−1.37	−2.05^*^ (adj. *p* = 0.240)	0.55
Increasing low SE	9	Raw		14	16	21	19	20.30^***^ (3, 9)	−1.34	−1.83	0.09
		Z-score	*Md*	−1.27	−1.2	−0.91	−1.1	2.47 (3, 9)	NA	NA	NA
Low SE	18	Raw		19	22	26	25	20.30^***^ (3, 18)	−1.87	−1.94^*^ (adj. *p* = 0.317)	0.19
		Z-score	*Md*	−1.17	−1.04	−0.95	−1.02	4.47 (3, 18)	NA		NA

* *p < 0.05,*

** *p < 0.01,*

*** *p < 0.001*.

**Table 7:** Effect sizes for changes in calculation fluency (RQ3).

**Group**	***N***	**Effect sizes (** ***r)***			
			**Pre1**	**Pre2**	**Post**
			**vs. Pre2**	**vs. Post**	**vs. Follow-up**
**High SE**	15	Raw	0.27	**0.63**	0.12
		Z-score	0.16	**0.57**	0.31
**Low-to-high SE**	16	Raw	0.33	0.44	0.08
		Z-score	0.24	0.36	0.10
**Increasing low SE**	9	Raw	0.32	0.43	0.02
		Z-score	NA	NA	NA
**Low SE**	18	Raw	0.31	0.32	0.03
		Z-score	NA	NA	NA

*Large effect sizes are written in bold. Following intervals for r were used according to Cohen (1988): no effect < 0.1; small effect 0.1–0.3; intermediate effect 0.3–0.5; >0.5 large effect*.

When comparing the gain scores in calculation fluency during the intervention using the Kruskal-Wallis test, statistically significant differences were found [χ^2^(59, 3) = 8.00, *p* = 0.047]. The paired comparison revealed that the high SE group improved more in calculation fluency during the intervention than the low SE group (*z* = 2.68, *p* = 0.007), and this finding remained when taking into account the Bonferroni correction for multiple tests (*p* = 0.043). No other statistically significant differences were found between the SE-profile groups.

## Discussion

This study extended previous research by comparing whether children's math self-efficacy (math SE) can be supported by a pure calculation strategy training or whether explicit SE support targeting the four sources of self-efficacy introduced by social-cognitive theory (Bandura, 1997) integrated with strategy training has added benefits for children's math SE, sources of math SE, and their calculation fluency. Special education teachers implemented these interventions at schools for children with poor calculation skills. First, this study examined how math SE changed among children who participated in calculation strategy training either with (SE group) or without explicit SE intervention (skill group). Second, changes in the four source experiences (mastery experiences, social persuasions, vicarious experiences, and emotional and physiological states) were examined by comparing the two intervention groups (SE and skill groups). Also, the relationship between the changes in the SE-source experiences and changes in SE beliefs was analyzed. Third, we examined how children with different SE levels (i.e., SE profiles formed based on the pre-intervention SE level and changes during the interventions) improved in calculation fluency.

The results showed, first, that low-performing children's math SE, that is, their beliefs about their capability to do and learn math, improved in both intervention groups (the SE and skill groups). However, only the intervention that combined strategy training and math-SE support enhanced math SE for children with low SE. Second, both interventions strengthened mastery experiences and lowered experiences related to negative emotional and psychological states. However, experiences of social persuasions increased only among children in the SE group. Moreover, changes in mastery experiences and social persuasions were positively associated with changes in math SE only among children who received explicit SE intervention (SE group). Third, a high level of math SE was related to positive development in calculation fluency during strategy training; children with high SE and children whose SE changed during the intervention from low to high SE showed more skill development, approaching their average grade level. In contrast, children with low SE did not reach their age peers in calculation fluency.

### Changes in Math-SE During Interventions

The findings showed a significant increase in math SE in both intervention groups (SE and skill) but not among the control group, suggesting that the changes in math SE were due to the provided interventions. This interpretation was further supported by the fact that changes in math SE took place during the intervention period and no change in math SE was found during the baseline or follow-up. Thus, it seems that providing individually challenging but accessible tasks and targeted strategy training can increase math SE in addition to improving calculation fluency itself (Koponen et al., 2018). There are several possible reasons for these positive effects. First, both interventions provided opportunities to practice and to perform successfully in math tasks despite difficulties, which is not the situation in business-as-usual instruction where educational plans are often followed and tasks are not tailored to the child's skill level. Moreover, it is possible that implementing the training in small groups with peers having similar skill levels might have lowered the excitement and nervousness related to expectations for performance and worry about failing in math.

Although improvement in math SE was found among both intervention groups, the level of math SE for children with initially low math SE changed during the interventions only in the SE group, in which a large effect size was found. It seems that the students most in need of support—for both poor mathematical skills and low math SE—need explicit SE support to be able to see their progress and change their beliefs about their own math skills. Skill training itself was not found to be sufficient to change math SE among these children, although changes in skills were found. Thus, our findings provide empirical support for the theory-derived assumption that by enabling positive source experiences through explicit SE intervention, it is possible to enhance children's SE. This finding of the malleability of math SE aligns with previous results of self-efficacy research in other academic domains, that is, in reading (Aro et al., 2018) and in writing (García and de Caso, 2006). These findings further support the effectiveness of source-based SE interventions, especially for children with low SE, and highlight the importance of integrating explicit self-efficacy feedback and practices into instruction provided at schools.

### Changes in Source Experiences and Their Relation to Changes in SE During Interventions

In general, children in both intervention groups experienced more positive source experiences (i.e., total score of the four sources) after the interventions. A more detailed analysis of each of the source experiences revealed that children reported more mastery experiences and fewer negative arousals (i.e., emotional and psychological states) after the interventions across both intervention groups. The findings suggest that calculation strategy training administered in small groups of students with similar difficulties and using tasks with an appropriate difficulty level provides students with opportunities both to experience mastery in tasks and to decrease the negative emotional reactions related to math. These results are in line with suggestions that mastery experiences can be provided by using individually challenging but accessible tasks (Bandura, 1994). In everyday school life, many children who have problems with basic calculation skills have the same educational aims and curricula as their peers who do not have problems learning math. Following the same instructions and facing daily challenges at school are not likely to provide mastery experiences but, instead, lead to experiences of failure which can influence on emotional and psychological states as well. However, if students experience only easy successes, it could lead them to expect quick results and become easily discouraged by failure (Bandura, 1994), and thus, individual learning plans with individually adjusted challenge levels are important in educational support. The element of an appropriate difficulty level of tasks was present in both intervention groups. A smooth decrease found in emotional and psychological states in both intervention groups could be explained by a sense of mastery created from appropriately difficult math tasks, which could have reduced the negative emotions toward math, such as math anxiety, that has been shown to be dependent on task difficulty (Pantoja et al., 2020). In support of this interpretation, psychological state and mastery experiences were found to correlate strongly in a study that examined the sources of SE in math among middle school students (Usher and Pajares, 2009).

Changes in mastery experiences were positively associated with changes in math SE among the SE group. Mastery experience has previously been found to relate to math SE among third grade elementary school students (Joët et al., 2011), and our results extended these previous cross-sectional findings by providing stronger evidence of associations and confirming theoretical assumptions that mastery experiences are central sources of math SE. Both intervention groups exhibited positive growth in mastery experiences during the interventions; however, changes in mastery experiences were related more strongly to changes in math SE among the SE group. One explanation for this finding might be that children in the SE group received feedback and were involved in practices that explicitly guided them in making interpretations and linking experiences of success and mastery to their capability to do and learn math. This is in line with the claim that the effect of successful performance on SE varies according to how various personal and situational contributions are interpreted and weighted (Bandura, 1997). This notion implies that a teacher can promote and support a child's individual interpretation of their successful performances and help the child to see these experiences as signs of their capability to successfully learn or perform math in the future. Performance accomplishments may not automatically lead to mastery interpretation or add confidence to one's capability in math. Rather, this is something that the teacher can and should explicitly support, for instance, by making the child's progress visible to the child and highlighting the interpretation that improvement is a result of the child's practice, which demonstrates his or her capability to learn math.

Only children in the explicit SE intervention (SE group) experienced increasing social persuasions over time. In addition, change in social persuasions was positively associated with change in math SE only in the SE group. It was somewhat surprising to find an increase only in the SE group, because, at first glance, social persuasions could be considered a rather self-evident element of teaching and general instruction. Our findings support the positive effects of explicit positive feedback given for effort on training and skill development as well as encouragement of the group members to provide positive feedback for each other. It seems that although children likely receive verbal persuasions of their skills in normal teaching practices, students experience teacher support as more persuasive when teachers are instructed and guided to give more explicit and positive feedback on students' progress and efforts. The teachers were instructed to provide feedback on progress, success, and effort systematically during each training session, which might strengthen the experiences of being praised. In addition to the teacher's verbal persuasions, children were also encouraged to pay attention and praise others' learning and improvement; children in the SE group received persuasions from other children more frequently than those in the skill group (see below). This might not be a typical part of the business-as-usual support, where social persuasion is mainly received from teachers. Thus, this could be a significant factor to consider when developing learning environments supporting SE. It may also be that when children learn to see the progress and effort of their peers and to encourage them, they may learn to recognize their own progress and efforts and praise themselves (Pajares, 2006).

The finding that change in social persuasions is positively associated with changes in math SE among the SE group is in line with the proposal that particularly younger students use the persuasions received from others when forming beliefs of their own capabilities (Bandura, 1997) and in line with recent findings in reading showing that these experiences shape SE development (Peura et al., 2021). The social persuasion provided in the SE intervention was more explicit and systematically provided than the spontaneous positive feedback children likely have received at school. The teachers were instructed to give self-referenced feedback and focus on self-improvement rather than on triumph over others (Bandura, 1997). Moreover, the feedback was targeted to help the child to see their progress and focus on improvement, no matter how small that improvement might have been. In prior studies, students who received self-referenced feedback were shown to have higher SE than those who received other-referenced feedback or norm-referenced feedback (Shih and Alexander, 2000; Chan and Lam, 2008). This is an important and encouraging finding that clarified important features of feedback for enhancing SE. As Bandura (1997) has emphasized, social persuasion does not obviously enhance SE, and it may actually be even easier to undermine rather than enhance an individual's SE through social persuasions. Social persuasions should focus on self-improvement rather than on triumph over others (Bandura, 1997). This understanding may be especially needed for encouraging low-performing students who may experience disappointing results despite their efforts and constant struggle with learning. Moreover, social persuasions should be realistic because unrealistic boosts in efficacy are quickly disconfirmed by disappointing results from one's efforts (Bandura, 1997; Pajares, 2006). These issues were emphasized in the present SE interventions.

To ensure that interventions were implemented as planned, the children completed fidelity ratings for questions on how often they experienced practices and feedback that were planned to provide experiences in the four sources of SE. The children in the SE group reported more actions related to all four source experiences, but no differences were found in general features concerning the intervention atmosphere and content. This finding suggests that the SE intervention was implemented as planned to cover all four sources of SE. This was encouraging, since it emphasized the ecological validity of the present study by providing research evidence for SE intervention programs that can be implemented rather easily in schools.

The heterogeneity found in the changes of the four source experiences as well as in their association with SE were not likely due to fidelity issues, because expected differences between two intervention groups were systematically found in practices and feedback targeted at providing positive experiences in all four sources. Thus, this study provided new knowledge regarding the malleability of sources of SE by showing that, at least among elementary school children, source experiences differed according to how easily they could be changed (i.e., malleability). Moreover, the source experiences seemed to be differently weighted in relation to SE, as has been suggested to occur among older children (Chen and Usher, 2013). Among elementary school children, mastery experiences and social persuasions seemed to be the most relevant efficacy-building experiences in math.

### Differences in the Level and Changes in SE and Calculation Fluency Improvement During Interventions

Finally, we focused on whether the differences in the level and changes of math-SE were visible in calculation fluency improvement during intervention. Children from both intervention groups were classified into four different source SE profiles: high SE, low-to-high SE, low-increasing SE, and low SE. The results indicated that children with high SE before and after the intervention developed the most in calculation fluency during the intervention, and they also approached their average grade level as indicated by the analyses using standard scores. These findings align with those of previous longitudinal studies (Pajares and Graham, 1999; Phan, 2012b), in which the level of SE was found to predict later math performance. Similar findings were also made in reading that showed that children with high SE benefit more from skill training than those with low SE (Ronimus et al., 2020). As children with high SE are suggested to put forth more effort and persistence in learning situations and to choose more learning activities (Bandura, 1986, 1997), it is not surprising that they also improved more, as was shown in our study. Children who changed from low to high SE also improved in calculation fluency and approached their average grade level during the interventions. This finding does not allow a causal conclusion of the unidirectional relations (i.e., that increasing self-efficacy boosted skill development). Rather, alternative interpretations that improvement in math achievement boosted math SE or there were reciprocal influences are possible. Reciprocal interactions between self-efficacy and achievement are supported in social-cognitive theory.

Children with low SE before and after the interventions showed significant development in calculation fluency during the interventions but did not approach the average grade level. The differences in the findings using raw scores and z-scores during the interventions can be explained by the fact that the low SE group mainly included older children (although not solely), and although there was improvement in calculation fluency during the intervention, it was not large enough to change their position within the distribution in the grade. Thus, by exploring both raw scores and z-scores, we obtained a more comprehensive picture of how calculation skills improved when grade-level expectations were taken into account.

Finally, children with a higher but still low math SE before and after the intervention (low-increasing SE) showed improvement in calculation fluency during the interventions but did not approach their average grade level during the interventions. It would have been interesting to see whether a longer intervention would have led to a stronger increase in both skills and SE because there was a smooth but positive trend for both self-efficacy and calculation fluency development.

Altogether, these findings support the view that high SE is related to stronger improvement in learning, and because math SE was shown to be malleable with the interventions provided, it is relevant to take it into account as a specific area of support at school and home. Our findings challenge the results from a recent meta-analytic study (Talsma et al., 2018) that suggests unilateral relations from achievement to SE among children, and instead, emphasize that high SE forms a stronger basis for learning among elementary school children, and thus, children's positive self-efficacy beliefs should be included as an important pedagogical aim in teaching along with objectives related to academic achievement and learning. However, the finding indicating that high SE did not boost the calculation fluency development during the baseline or follow-up highlights the importance of systematic, intensive, and continuous support for SE and of targeted strategy training for poor-performing children. Thus, low-performing children need ongoing support. An integrated approach that combines strategy training and SE intervention seems beneficial, especially among children with low calculation fluency and low math SE.

### Limitations and Directions for Future Research

Some limitations of this study should be considered when interpreting the findings. The main limitations were related to the quasi-experimental nature of the design. To emphasize the high societal value, this study was implemented in ecologically valid conditions by teachers as part of the everyday school routine; thus, a blinded and fully random matching of the participants was not possible. Moreover, the children were carefully selected for interventions, and because of the randomization at the school level, the SE and skill groups did not differ in calculation fluency in pre-assessments. However, the level of SE was not controlled for when matching, and there was a large variation from low to high SE in both intervention groups. A larger sample would have made it possible to analyze the individual variation in more detail. Moreover, because of the moderate sample sizes, it was not possible to analyze the findings for boys and girls separately, although gender might moderate the effects of the interventions on both source experiences and math SE (see Chen and Usher, 2013). Because of the limited available resources, procedures that would allow closer monitoring of the reliability and validity of the interventions (e.g., video recordings) could not be conducted. The measures taken to guarantee the fidelity of the results (teacher training, a session-by-session manual, diary completion, meetings, and phone calls during the intervention) support the assertion that the programs were implemented following the program manual and intervention design.

Moreover, in the present study we used Bandura's socio-cognitive theory as a theoretical frame. For the sake of clarity we did not introduce related and partially overlapping concepts, such as math anxiety (compare to physiological and emotional state). However, math anxiety is important factor and related both to self-efficacy as well as skill development (e.g., Sorvo et al., 2019). In future, intervention studies should include, the broader set of items representing the different dimensions of math anxiety, such as cognitive and affective (e.g., Ho et al., 2000; Sorvo et al., 2019) in order to exam the interaction with math-SE and math anxiety more deeply. Furthermore, by using person oriented approaches it's possible to clarify the predictive relation of these intertwined factors by examining individual profiles formed across these emotional and motivational factors and their relation with skill development or response to support. Moreover, SE beliefs are linked to child's behavior and self-regulation in learning situation (Bandura, 1986, 2001) as well as to metacognitive skills (Cera et al., 2013) which were not examined in the present study. In addition to self-regulation, also external regulation stemming from the context is relevant especially in group-based interventions as the context may or may not promote positive proactivity and foster regulation. Thus, they are relevant factors to consider in future research when trying to understand the link between SE and skill development. Finally, intervention were implemented in small groups, but as a limitation, information of interactions among children or with teacher were not collected, and thus, the effect of these factors were not explored.

### Practical Implications

There are several practical implications. The explicit intervention that targeted the four sources of self-efficacy, integrated with intensified strategy training and implemented by teachers in small groups, was effective in building positive self-beliefs and positive efficacy experiences as well as increasing math skills. This suggests that feedback that highlights the self-monitoring of progress and personal accomplishments is well-suited for building a sense of efficacy, which in turn promotes math achievement. The present study showed that children with low calculation fluency and low math SE did not benefit from pure strategy training to the same extent as children with low calculation fluency but high math SE. More importantly, the most vulnerable children, those with low math SE and low skills, seemed to benefit from explicit SE support. Thus, in addition to identifying children who have a low skill level and are therefore in need of intensified training, it is important to identify a child's level of SE and understand how it influences the child's behavior, such as persistence and effort in learning situations. Providing mastery experiences and social persuasions seem to be promising approaches to enhance math SE among elementary school children. Most importantly, the SE intervention program was integrated with skill training and implemented by special education teachers as part of their normal work to support low-performing children; thus, it can be directly applied at schools.

## Data Availability Statement

The datasets presented in this article are not readily available because written consent from parents allow only research group members to analyze and publish data. Requests to access the datasets should be directed to tuire.k.koponen@jyu.fi.

## Ethics Statement

The studies involving human participants were reviewed and approved by University of Jyväskylä Ethical Committee. Written informed consent to participate in this study was provided by the participants' legal guardian/next of kin.

## Author Contributions

All co-authors have made contributions appropriate for assumption of authorship and were in agreement with the content of the manuscript and byline order.

## Conflict of Interest

The authors declare that the research was conducted in the absence of any commercial or financial relationships that could be construed as a potential conflict of interest.

## Publisher's Note

All claims expressed in this article are solely those of the authors and do not necessarily represent those of their affiliated organizations, or those of the publisher, the editors and the reviewers. Any product that may be evaluated in this article, or claim that may be made by its manufacturer, is not guaranteed or endorsed by the publisher.
