# Supplementary material for: Benefits of Integrating an Explicit Self-Efficacy Intervention With Calculation Strategy Training for Low-Performing Elementary Students
Source: Front Psychol. 2021 Aug 6;12:714379. doi: 10.3389/fpsyg.2021.714379 (PMC8377810; doi:10.3389/fpsyg.2021.714379)
Supplement: Supplementary file 1 [file Table_1.DOCX]

Appendix A.

*Math Self-Efficacy Scale*

| **Original items in Finnish** | **Translated items** |
| --- | --- |
| *How certain are you that you can…* | |
| ***Items related to capability in math*** | |
| … tekemään kaikki matematiikan kotitehtävät, vaikka ne olisivat sinulle vaikeita? | …do all math assignments, although you find them difficult? |
| ...laskemaan yhteenlaskuja nopeasti mielessä? | …calculate sums fast in mind? |
|  |  |
| ***Items related to developing better math skill*** | |
| ...oppimaan laskemaan yhteenlaskuja nopeammin? | …learn to calculate faster? |
| ...oppimaan laskemaan niin, että teet vähemmän virheitä? | …learn to calculate so that you make fewer mistakes? |
|  |  |
| ***Items targeting daily activities requiring math*** | |
| ...laskemaan kaupassa, kuinka paljon sinulla on rahaa? | …calculate in store how much money you have? |
| ...laskemaan kaupassa, riittävätkö rahasi ostoksiin tai kuinka paljon saat rahaa takaisin? | …calculate in store do you have enough money and how much you should get back? |
| ...pelaamaan lautapelejä, joissa pitää laskea päässä nopeasti? | …play board games, where you have to calculate fast in mind? |

Appendix B.

*Sources of Math Self-Efficacy Scale*

| **Original items in Finnish** | **Translated items** | **Original items (Usher & Pajares, 2009)** |
| --- | --- | --- |
| ***Mastery experience*** | |  |
| Olen aina ollut hyvä matematiikassa. | I have always been good with math. | I have always been successful with math. |
| Osaan matematiikkaa hyvin. | I do well on math. | I do well on math assignments. |
| Osaan laskea hyvin vaikeitakin matematiikan tehtäviä. | I do well on even the most difficult math assignments. | I do well on even the most difficult math assignments |
| ***Social persuasion*** | |  |
| Opettajani on usein kehunut siitä, että matematiikan taitoni ovat parantuneet. | My teacher has often told that I am getting better in math. | My math teachers have told that I am good at learning math. |
| Matematiikan taitoani on usein kehuttu. | I have been praised for my math skills. | I have been praised for my ability in math. |
| Luokkakaverit ovat sanoneet, että olen hyvä matematiikassa. | My classmates have told me that I'm a good in math. | Other students have told me that I’m good at learning math. |
| ***Vicarious experience*** | |  |
| Kun näen toisten lasten pärjäävän matematiikassa minua paremmin, se saa minutkin opettelemaan matematiikkaa. | Seeing kids do better than me in math pushes me to do better. | Seeing kids do better than me in math pushes me to do better. |
| Ajattelen usein, että jonain päivänä olen taitava laskija. | I often imagine myself being a good in calculation. | I imagine myself working through challenging math problems successfully. |
| Ihailen aikuisia, jotka ovat hyviä matematiikassa. | I admire adults who are good in math. | (not in the original scale) |
| ***Physiological and emotional states*** | |  |
| Ahdistun, kun tiedän, että joudun vastaamaan matematiikan tunnilla. | I get anxious when I know that I have to give a answer in math class. | (not in the original scale) |
| Ahdistun, kun aloitan matematiikan tehtäviä. | I start to feel anxious as soon as I begin to do math assignments. | I start to feel stressed-out as soon as I begin my math work. |
| Tunnen kehossani jännitystä, kun minun pitää tehdä matematiikan tehtäviä. | I feel tension in my body when I have to do math assignments. | My whole body becomes tense when I have to do math. |
| *Note.* The original items (Usher & Pajares, 2009) were translated from English to Finnish and then adapted for the Finnish primary school children. The items were then back-translated to English. | | |
